# The impact of preoperative biliary drainage on postoperative healthcare-associated infections and clinical outcomes following pancreaticoduodenectomy: a ten-year retrospective analysis

**DOI:** 10.1186/s12879-024-09246-8

**Published:** 2024-03-28

**Authors:** Zheng-Hao Yu, Ming-Mei Du, Xuan Zhang, Ji-Jiang Suo, Tao Zeng, Xiao-Lian Xie, Wei Xiao, Qing-Bin Lu, Yun-Xi Liu, Hong-Wu Yao

**Affiliations:** 1 Department of Disease Prevention and Control, The First Medical Center, Chinese PLA General Hospital, 28 Fu-Xing Road, Haidian District, Beijing, 100853 P. R. China; 2 Medical School of Chinese PLA, Beijing, P. R. China; 3 Faculty of Hepato-Pancreato-Biliary Surgery, Chinese PLA General Hospital, Beijing, P. R. China; 4 Institute of Hepatobiliary Surgery of Chinese PLA, Beijing, P. R. China; 5 Department of Central Sterile Supply, Ningxia People’s Armed Police Corps Hospital, Yinchuan, P. R. China; 6 Department Of Hospital Infection-Control, Lanzhou University Second Hospital, Gansu, P. R. China; 7 Department of Laboratorial Science and Technology, School of Public Health, Peking University, 38 Xue-Yuan Road, Haidian District, Beijing, 100191 P. R. China

**Keywords:** Healthcare-associated infections, Pancreaticoduodenectomy, Preoperative biliary drainage, Clinical outcomes

## Abstract

**Background:**

Pancreaticoduodenectomy (PD) is a complex procedure and easily accompanied by healthcare-associated infections (HAIs). This study aimed to assess the impact of PBD on postoperative infections and clinical outcomes in PD patients.

**Methods:**

The retrospective cohort study were conducted in a tertiary hospital from January 2013 to December 2022. Clinical and epidemiological data were collected from HAIs surveillance system and analyzed.

**Results:**

Among 2842 patients who underwent PD, 247 (8.7%) were diagnosed with HAIs, with surgical site infection being the most frequent type (*n* = 177, 71.7%). A total of 369 pathogenic strains were detected, with *Klebsiella pneumoniae* having the highest proportion, followed by *Enterococcu* and *Escherichia coli*. Although no significant association were observed generally between PBD and postoperative HAIs, subgroup analysis revealed that PBD was associated with postoperative HAIs in patients undergoing robotic PD (aRR = 2.174; 95% CI:1.011–4.674; *P* = 0.047). Prolonging the interval between PBD and PD could reduce postoperative HAIs in patients with cholangiocarcinoma (≥4 week: aRR = 0.292, 95% CI 0.100–0.853; *P* = 0.024) and robotic PD (≤2 week: aRR = 3.058, 95% CI 1.178–7.940; *P* = 0.022). PBD was also found to increase transfer of patients to ICU (aRR = 1.351; 95% CI 1.119–1.632; *P* = 0.002), extended length of stay (*P* < 0.001) and postoperative length of stay (*P* = 0.004).

**Conclusion:**

PBD does not exhibit a significant association with postoperative HAIs or other outcomes. However, the implementation of robotic PD, along with a suitable extension of the interval between PBD and PD, appear to confer advantages concerning patients’ physiological recuperation. These observations suggest potential strategies that may contribute to enhanced patient outcomes.

**Supplementary Information:**

The online version contains supplementary material available at 10.1186/s12879-024-09246-8.

## Introduction

Pancreaticoduodenectomy (PD), a complex operation for the treatment of pancreatic cancer and other related diseases [1], has been widely applied with the increasing incidence of pancreatic cancer recently, particularly in developing countries [2]. Despite the advancements in medical technology reducing the mortality rate of PD patients to below 5%, postoperative complications continue to pose significant challenges [3–8]. As one of the postoperative complications in patients after PD, healthcare-associated infections (HAIs) can result in poor medical quality, increasing medical cost and mortality [9–13]. Therefore, it is imperative to take effective measures to prevent their occurrence throughout the entire treatment process.

Preoperative biliary drainage (PBD) is often used to reduce obstructive jaundice in patients before PD. However, it remains disputable about the routine PBD for PD [14]. Some studies suggest that PBD can offers benefits such as improved liver functions, restoration of physiological mechanisms altered by obstructive jaundice, and increase surgical tolerance among patients [15–19]. Conversely, other studies have reported adverse postoperative consequences including postoperative pancreatic fistula, and even patient mortality [20–22]. Consequently, further investigation involving larger sample sizes is necessary to establish more robust evidence to explore the impact of PBD on HAIs and other outcomes following PD.

Given the ongoing debates surrounding previous studies, we have gathered a decade’s worth of data on PD for the purpose of this study. This research might offer valuable insights for the development of preventive and control measures.

## Material and methods

### Data collection

This retrospective cohort study was conducted at a 3800-bed tertiary hospital serving approximately 13,000 inpatients per month in Beijing, China. The data on the clinical and epidemiological characteristics of patients undergoing PD during 2013 to 2022 were collected from the real-time nosocomial infection surveillance system (RT-NISS) [23]. The diagnosis and confirmation of HAI cases were conducted within the RT-NISS by qualified infection prevention specialists according to the Chinese Nosocomial Infection Diagnosis Criterion (2001) issued by the National Health Commission of China [24]. All sensitive information of patients was excluded in this study, and ethical approval was obtained from the Medical Ethics Committee (approval number: S2019-142-02).

The data includes the following: 1) demographic characteristics: age, sex, height, weight and body mass index (BMI); 2) clinical data: history of smoking, history of drinking, comorbidities (hypertension, diabetes and coronary heart disease), date of admission and discharge; 3) perioperative characteristics: total number of operations, the name and type of each operation, whether PBD was conducted or not, type of PBD, and the interval between PBD and PD; 4) outcome: type of HAI, date of HAI occurrence, the pathogens responsible for the HAIs, the other presence of postoperative complications such as postoperative pancreatic fistula, biliary leakage, delayed gastric emptying and postoperative pancreatitis, transfer of patients to ICU, mortality, the length of stay (LOS) and postoperative LOS.

### Inclusion and exclusion criteria

**Fig. 1:**
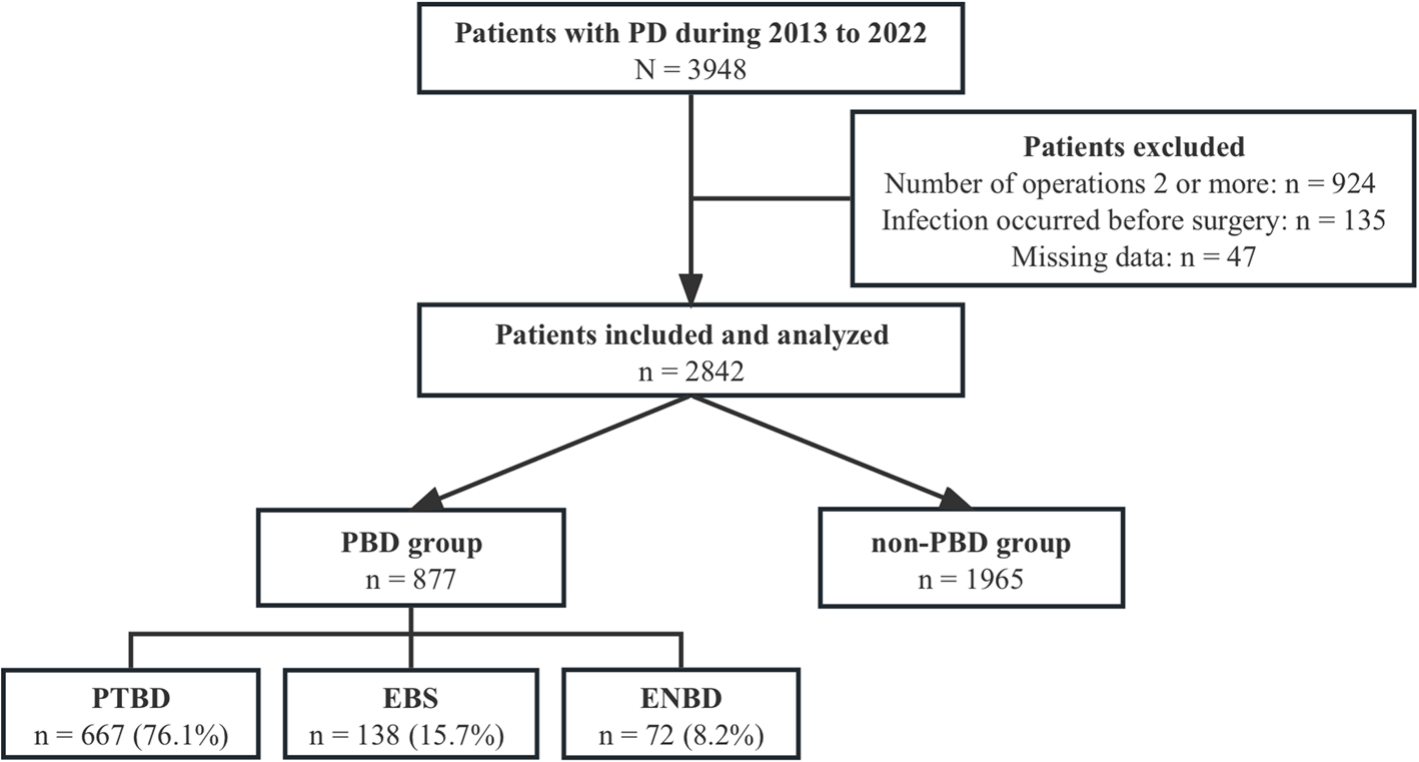
Flowchart of the included participants. PD, pancreaticoduodenectomy; PBD, preoperative biliary drainage; PTBD, percutaneous transhepatic biliary drainage; EBS, endoscopic biliary stenting; ENBD, endoscopic nasobiliary drainage

Inclusion criteria are as follows: 1) Patients who underwent PD and were admitted between January 1, 2013 and December 31, 2022; 2) Hospitalization duration exceeding 2 days; 3) Only one PD operation performed; 4) Patients had no history of HAIs prior to PD. Exclusion criteria: 1) Patients who underwent two or more operations during hospitalization; 2) HAIs occurring before PD; 3) Patients with missing information. Finally, 2842 patients were enrolled (Fig. 1).

### Definitions

PBD procedures encompassed three main types: percutaneous transhepatic biliary drainage (PTBD), endoscopic biliary stenting (EBS), and endoscopic nasobiliary drainage (ENBD) [25]. The patients’ medical records were reviewed to determine whether they had undergone PBD, and the type of PBD performed was categorized as PTBD, EBS, or ENBD. Additionally, the interval between PBD and PD was further classified into three temporal segments: ≤1 week, 1–4 weeks, and ≥ 4 weeks.

The primary postoperative complications of patients undergoing PD were defined using criteria consistent with previous studies [26–28], including postoperative pancreatic fistula (POPF), biliary leakage, delayed gastric emptying (DGE), and postoperative pancreatitis. Furthermore, transfer of patients to ICU, mortality, LOS and postoperative LOS were considered as secondary outcomes.

### Statistical analysis

The sample size was calculated using PASS software. A minimum of 237 participants in PBD group and non-PBD group were calculated with the power at 90% along with a two-sided significance level of 0.05.

Descriptive analyses were conducted to summarize the data. Continuous variables were reported as means with standard deviation or median with interquartile ranges (IQR), while categorical variables were presented as frequencies and proportions. To assess the impact of individual variables and identify potential factors associated with HAIs among patients after PD, Chi-square tests, two sample t-tests and Wilcox rank sum test were performed. Pearson correlation coefficient was used to show the correlation between pancreatic fistula and infection. The actual effect of PBD was determined by adjusted for other covariates, and the confounders of age, gender, BMI, smoking, drinking, hypertension, diabetes, coronary disease, primary disease and operation approach were adjusted in the multivariate analysis. The binary logistic regression model was used to estimate the effects of PBD on postoperative HAIs and complications in patients after PD, presenting risk ratios (RR) or adjust risk ratios (aRR) along with 95% confidence interval (CI). The generalized linear regression model was employed if the outcome variable was continuous. Statistical significance was set at the 5% level. All statistical analyses were carried out using R software (version 3.6.3).

## Results

### Epidemiological characteristics

Among 2842 patients included in this study, a total of 877 (30.9%) underwent PBD, while the remaining 1965 (69.1%) patients were assigned to the non-PBD group (Fig. 1). Of the total patients, 1738 (61.2%) were males and 1104 (38.8%) were females (Table 1). The mean age was 58.1 (±SD 11.5) years, with 879 (30.9%) of them being 65 years or older. Among the study participants, pancreatic cancer (831, 29.2%) was the most common primary disease, followed by cholangiocarcinoma (640, 22.5%), duodenal carcinoma (438, 15.4%), ampullary carcinoma (217, 7.6%), intraductal papillary mucinous neoplasm (IPMN) (176, 6.2%), and other diseases (540, 19%). 2013 (70.8%) patients underwent open surgery, 619 (21.8%) received a robotic approach, 163 (5.7%) underwent a laparoscopic procedure, and 47 (1.7%) had a combination of robotic and laparoscopic surgery. Patients with PBD were older (60.0 vs. 57.2 years; *P* < 0.001), more male (65.5% vs. 59.2%; *P* = 0.001) and had lower BMI (23.0 vs. 23.5 Kg/m^2^; *P* < 0.001). The PBD group had a higher proportion of cholangiocarcinoma (37.5% vs. 15.8%; *P* < 0.001) and fewer robotic approach (18.4% vs. 23.3%; *P* = 0.007) than the non-PBD group. And there was a positive correlation between POPF and postoperative infection (r = 0.105, *P* < 0.001).

**Table 1:** Demographic and clinical characteristics of the study participants

Characteristics	Total participants (*N* = 2842)	PBD group	Non-PBD group	*P* value	Infection	Non-infection	*P* value
		(*n* = 877)	(*n* = 1965)		(*n* = 247)	(*n* = 2595)	
**Age, years, mean ± SD**	58.1 ± 11.5	60.0 ± 10.2	57.2 ± 12.0	< 0.001	58.6 ± 11.1	58.0 ± 11.6	0.47
**Age, years, n (%)**							
< 65	1963(69.1)	567(64.7)	1396(71.0)	0.001	168(8.6)	1795(91.4)	0.707
≥65	879(30.9)	310(35.3)	569(29.0)		79(9)	800(91)	
**Gender, n (%)**							
Male	1738(61.2)	574(65.5)	1164(59.2)	0.002	158(9.1)	1580(90.9)	0.342
Female	1104(38.8)	303(34.5)	801(40.8)		89(8.1)	1015(91.9)	
**BMI, Kg/m**^**2**^**, mean ± SD**	23.4 ± 3.3	23 ± 3.2	23.5 ± 3.3	< 0.001	24.3 ± 3.4	23.3 ± 3.3	< 0.001
**BMI, Kg/m**^**2**^**, n (%)**							
< 18.5	167(5.9)	66(7.5)	101(5.1)	0.001	7(4.2)	160(95.8)	0.004
18.5–24	1513(53.2)	495(56.4)	1018(51.8)		117(7.7)	1396(92.3)	
24–27	772(27.2)	218(24.9)	554(28.2)		75(9.7)	697(90.3)	
≥27	390(13.7)	98(11.2)	292(14.9)		48(12.3)	342(87.7)	
**Smoking, n (%)**	843(29.7)	268(30.6)	575(29.3)	0.485	72(8.5)	771(91.5)	0.854
**Drinking, n (%)**	827(29.1)	269(30.7)	558(28.4)	0.217	81(9.8)	746(90.2)	0.181
**Hypertension, n (%)**	719(25.3)	215(24.5)	504(25.6)	0.521	72(10)	647(90)	0.145
**Diabetes, n (%)**	542(19.1)	166(18.9)	376(19.1)	0.897	43(7.9)	499(92.1)	0.487
**Coronary disease, n (%)**	244(8.6)	71(8.1)	173(8.8)	0.534	23(9.4)	221(90.6)	0.67
**Primary disease, n (%)**							
Pancreatic cancer	831(29.2)	253(28.8)	578(29.4)	< 0.001	55(6.6)	776(93.4)	0.001
Cholangiocarcinoma	640(22.5)	329(37.5)	311(15.8)		76(11.9)	564(88.1)	
Duodenal carcinoma	438(15.4)	112(12.8)	326(16.6)		44(10)	394(90)	
Ampullary carcinoma	217(7.6)	116(13.2)	101(5.1)		23(10.6)	194(89.4)	
Intraductal papillary mucinous neoplasm (IPMN)	176(6.2)	11(1.3)	165(8.4)		6(3.4)	170(96.6)	
Others	540(19.0)	56(6.4)	484(24.6)		43(8)	497(92)	
**Operation approach, n (%)**							
Open	2013(70.8)	638(72.7)	1375(70)	0.007	194(9.6)	1819(90.4)	0.019
Robot	619(21.8)	161(18.4)	458(23.3)		36(5.8)	583(94.2)	
Laparoscope	163(5.7)	63(7.2)	100(5.1)		15(9.2)	148(90.8)	
Robot+laparoscope	47(1.7)	15(1.7)	32(1.6)		2(4.3)	45(95.7)	

*PBD* preoperative biliary drainage, *BMI* Body Mass Index

**Fig. 2:**
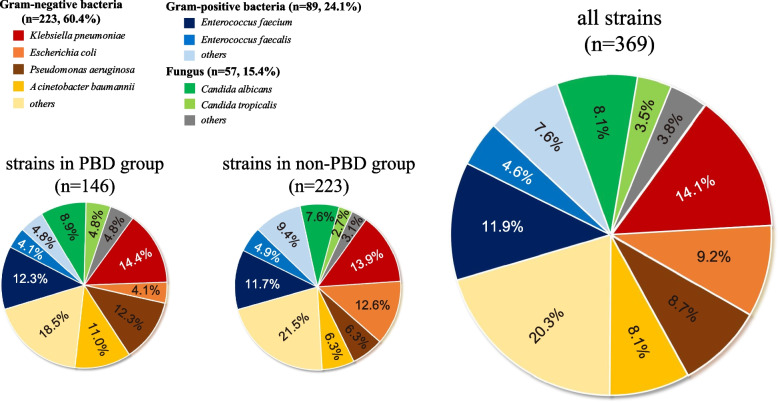
Pathogens distribution in HAI patients with PD. PD, pancreaticoduodenectomy; PBD, preoperative biliary drainage; HAIs, healthcare-associated infections

During 2013–2022, a total of 247 patients (8.7, 95% CI: 7.7%–9.8%) developed HAIs following PD. Of these cases, surgical site infections (SSI) accounted for the majority at 177 (71.7%), followed by bloodstream infections (42, 17.0%), lower respiratory tract infection (16, 6.5%), gastrointestinal system infection (7, 2.8%), and others (5, 2.0%). Patients with robotic PD and lower BMI had lower rates of postoperative HAIs (all *P* < 0.05). A total of 369 pathogens were identified among 199 HAI patients, with Gram-negative bacteria (223, 60.4%) being the most prevalent, followed by Gram-positive bacteria (89, 24.1%) and fungi (57, 15.4%). The pathogen samples were mainly from abdominal drainage fluid (114, 57.3%), followed by venous blood (40, 20.1%) and bile (21, 10.6%). The most common pathogens causing postoperative HAIs in PD patients were *Klebsiella pneumoniae* (52, 14.1%), *Enterococcus faecalis* (44, 11.9%), and *Escherichia coli* (34, 9.2%) (Fig. 2). In the PBD group, the predominant pathogens were *Klebsiella pneumoniae* (21, 14.4%), *Pseudomonas aeruginosa* (18, 12.3%), and *Enterococcus faecalis* (18, 12.3%). Among the non-PBD group, the most frequently encountered pathogens were *Klebsiella pneumoniae* (31, 13.9%), *Escherichia coli* (28, 12.6%), and *Enterococcus faecalis* (26, 11.7%). The distribution of pathogens between the two groups did not show a statistically significant difference (χ^2^ = 2.287, *P* = 0.319).

### Influencing factors assessment and subgroup analyses

Patients in PBD group had a slightly higher incidence of postoperative HAIs compared to those without PBD, although the difference was not statistically significance (10.0% vs. 8.1%; *P* = 0.089). After adjusting for other covariates, no significant association was also found between PBD and the risk of postoperative HAIs (aRR = 1.112; 95% CI: 0.828–1.493; *P* = 0.48). Similarly, there were no significant associations observed between different types of PBD procedures, the interval between PBD and PD, and the risk of postoperative HAIs when adjusting for other variables (Table 2).

**Table 2:** Crude and adjusted analyses assessing the impact of PBD on postoperative HAIs

Variables	Infection	Non-infection	Crude analysis		Adjusted analysis	
	(*n* = 247)	(*n* = 2595)	RR, 95% CI	*P* value	aRR, 95% CI	*P* value
**PBD**						
No	159(8.1)	1806(91.9)	ref.		ref.	
Yes	88(10.0)	789(90.0)	1.267(0.964,1.665)	0.09	1.112(0.828,1.493)	0.48
**Type of PBD**						
Non-PBD	159(8.1)	1806(91.9)	ref.		ref.	
PTBD	68(10.2)	599(89.8)	1.289(0.957,1.738)	0.095	1.144(0.830,1.576)	0.412
EBS	11(8.0)	127(92.0)	0.984(0.520,1.860)	0.96	0.836(0.436,1.602)	0.588
ENBD	9(12.5)	63(87.5)	1.623(0.792,3.324)	0.186	1.402(0.674,2.913)	0.366
**Interval between PBD and PD**						
Non-PBD	159(8.1)	1806(91.9)	ref.		ref.	
≤1 week	20(12.0)	146(88.0)	1.556(0.949,2.552)	0.08	1.373(0.824,2.286)	0.224
1–4 week	40(9.2)	395(90.8)	1.150(0.800,1.654)	0.45	0.985(0.672,1.443)	0.937
≥4 week	28(10.1)	248(89.9)	1.282(0.840,1.958)	0.249	1.162(0.748,1.806)	0.504

*PBD* preoperative biliary drainage, *HAIs* healthcare-associated infections, *PTBD* percutaneous transhepatic biliary drainage, *EBS* endoscopic biliary stenting, *ENBD* endoscopic nasobiliary drainage, *RR* risk ratios, *RR* risk ratios, *aRR* adjusted risk ratios, *95% CI* 95% confidence interval

**Fig. 3:**
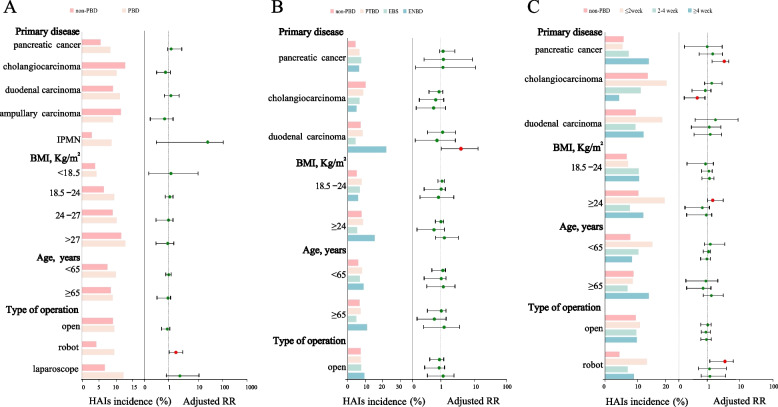
Subgroup analysis of assessing PBD for postoperative HAIs. **A** HAIs incidence and adjusted analyses assessing PBD for postoperative HAIs in subgroups. **B** HAIs incidence and adjusted analyses assessing type of PBD for postoperative HAIs in subgroups. **C** HAIs incidence and adjusted analyses assessing PBD-surgery interval for postoperative HAIs in subgroups. Groups whose sample size was too small to be included in the logistic regression analysis were excluded. In Fig. (3 B), we combined those with a BMI of 24–27 and ≥ 27 into BMI ≥24 because of the sample size. In Fig. (3 C), we divided the intervals under the robotic surgery subgroup into: ≤2 week ,2–4 week, and ≥ 4 week. PBD, preoperative biliary drainage; PTBD, percutaneous transhepatic biliary drainage; EBS, endoscopic biliary stenting; ENBD, endoscopic nasobiliary drainage; HAIs, healthcare-associated infections; BMI, Body Mass Index; RR, risk ratios

However, after adjusting for other covariates in the subgroup analyses, it was found that PBD increased the risk of postoperative HAIs in patients undergoing robotic PD (aRR = 2.174; 95% CI 1.011–4.674; *P* = 0.047) (Fig. 3A). When considering different types of PBD procedures, ENBD was associated with a higher postoperative HAIs incidence in patients suffering from duodenal carcinoma as their primary disease (aRR = 4.241; 95% CI 1.177–15.287; *P* = 0.027) (Fig. 3B). Among patients with pancreatic cancer, a PBD-PD interval of ≥4 weeks increased the risk of postoperative HAIs (aRR = 2.992; 95% CI 1.358–6.592; *P* = 0.007), whereas in patients with cholangiocarcinoma, PBD-PD interval of ≥4 weeks decreased the risk of postoperative HAIs (aRR = 0.292; 95% CI 0.100–0.853; *P* = 0.024). In patients with BMI > 24 Kg/m^2^, a PBD-PD interval of ≤1 week increased the risk of postoperative HAIs (aRR = 1.981; 95% CI 1.032–3.802; *P* = 0.04). Additionally, patients who underwent robotic PD had an increased risk of postoperative HAIs when PBD-PD interval was ≤2 weeks (aRR = 3.058; 95% CI: 1.178–7.940; *P* = 0.022) (Fig. 3C) (Table S1-S3).

**Fig. 4:**
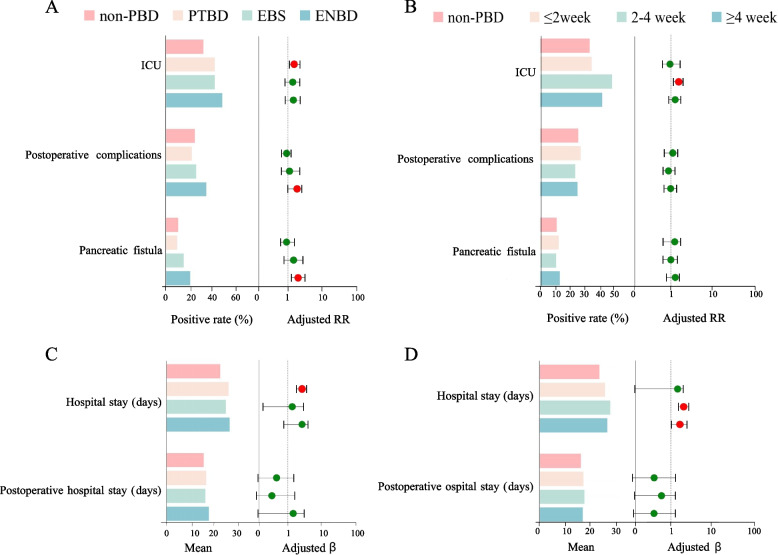
Logistic regression model and generalized linear regression model analysis for assessing the impact of type of PBD and PBD-surgery interval on postoperative outcomes in patients undergoing PD. **A** logistic regression model analysis for assessing the impact of type of PBD on postoperative outcomes in patients undergoing PD. **B** logistic regression model analysis for assessing the impact of PBD-surgery interval on postoperative outcomes in patients undergoing PD. **C** generalized linear regression model analysis for assessing the impact of type of PBD on postoperative outcomes in patients undergoing PD. **D** generalized linear regression model analysis for assessing the impact of PBD-surgery interval on postoperative outcomes in patients undergoing PD. Other clinical outcomes were not included in the regression analysis due to the small sample size. PD, pancreaticoduodenectomy; PBD, preoperative biliary drainage; PTBD, percutaneous transhepatic biliary drainage; EBS, endoscopic biliary stenting; ENBD, endoscopic nasobiliary drainage; ICU, Intensive Care Unit

Regarding clinical outcome, the PBD group exhibited several significant factors after adjusting for other covariates, including transfer to the ICU (aRR = 1.351; 95% CI: 1.119–1.632; *P* = 0.002), longer LOS (β = 3.475; 95% CI 2.644–4.306; *P* < 0.001) and longer postoperative LOS (β = 1.019; 95% CI 0.323–1.715; *P* = 0.004). However, there were no significant differences between the PBD group and the non-PBD group in terms of mortality, overall postoperative complications, or specific postoperative complications such as POPF, biliary leakage, and DGE (all *P* > 0.05) (Table 3). Furthermore, ENBD was significantly associated with overall postoperative complications (aRR = 1.691; 95% CI 1.018–2.809; *P* = 0.043) and pancreatic fistula (aRR = 2.181; 95% CI 1.187–4.007; *P* = 0.012) (Fig. 4A). Moreover, a PBD-PD interval of 1–4 week increased the risk of the transfer to ICU (aRR = 1.616; 95% CI 1.273–2.052; *P* < 0.001) (Fig. 4B). Regardless of the PBD method used, PTBD, EBS, and ENBD were significantly associated with longer LOS (all *P* < 0.05) (Fig. 4C). And there was a significant increase in LOS for PBD-PD intervals of 1–4 weeks and ≥ 4 weeks (P < 0.001) (Fig. 4D) (Table S4-S7).

**Table 3:** Crude and adjusted analyses assessing the impact of PBD on postoperative outcomes

Variables	PBD	Non-PBD	Crude analysis		Adjusted analysis	
	(*n* = 877)	(*n* = 1965)	RR, 95% CI	*P* value	aRR, 95% CI	*P* value
Transfer of patient to ICU, n (%)	373(42.5)	635(32.3)	1.567(1.328,1.847)	< 0.001	1.351(1.119,1.632)	0.002
Death, n (%)	11(1.3)	18(0.9)	1.375(0.647,2.924)	0.408	1.154(0.502,2.649)	0.736
Postoperative complications, n (%)	210(23.9)	489(24.9)	0.928(0.769,1.120)	0.437	0.968(0.790,1.187)	0.754
Pancreatic fistula, n (%)	100(11.4)	208(10.6)	1.051(0.813,1.358)	0.706	1.079(0.815,1.428)	0.595
Biliary leakage, n (%)	22(2.5)	59(3.0)	0.807(0.487,1.339)	0.407	0.899(0.522,1.549)	0.702
Delayed gastric emptying, n (%)	63(7.2)	141(7.2)	0.985(0.722,1.343)	0.924	0.995(0.712,1.391)	0.978

*PBD* preoperative biliary drainage, *ICU* Intensive Care Unit, *RR* risk ratios, *aRR* adjusted risk ratios, *95% CI* 95% confidence interval

## Discussion

This study retrospectively used long period and large sample data on the clinical and epidemiological characteristics of PD patients during 2013–2022 to explore the potential correlation between PBD and the risk of postoperative HAIs following PD. We comprehensively identified the epidemiological characteristics of patients with PD and observed that robotic-assisted PD and an optimal extended interval between PBD and PD appeared to confer benefits in reducing the occurrence of postoperative HAIs. Unlike previous studies with small samples, the data cases collected in this study were from a general hospital with numerous patients with PD admitted, and there was a sufficient sample to support the study.

PD is a complex procedure that would be susceptible to postoperative complications, including HAIs [29]. The overall postoperative HAIs incidence (8.7%) in our study was significantly lower the incidence than in other studies [11, 30]. Additionally, when comparing the rates of postoperative HAIs between different groups, we observed a lower incidence in both the PBD group (10.0%) and the non-PBD group (8.1%) compared to the incidences documented in a review conducted by Chen et al. (33.7% in the PBD group and 25.0% in the non-PBD group) [31]. This discrepancy can be attributed to several factors, including differences in the study participants, variations in the implementation of infection prevention and control measures, as well as variances in the proficiency of surgeons performing PD procedures. These factors have played a role in partially influencing the occurrence of postoperative HAIs and contributing to the slight increase in infection risk associated with PBD.

As reported in previous studies, *Klebsiella pneumoniae, Enterococcus* and *Escherichia coli* were the most common pathogens in postoperative HAIs populations who underwent PD in this study [32, 33]. This indicates the importance of targeted prophylactic antibiotic use in clinical practice, particularly focusing on these pathogenic organisms. Empirical administration of antibiotics to combat these organisms can be considered in cases of HAIs until the precise pathogen drug sensitivity are identified.

Obstructive jaundice is a common symptom among patients undergoing PD for pancreatic cancer and periampullary carcinomas [34–36]. It can lead to liver impairment, failure, and various clinical disturbances [37]. PD surgery in patients with obstructive jaundice may carry higher risks and increased postoperative complications [4, 38, 39]. For these special patients, PBD is necessary to alleviate the burden of jaundice before surgery, which plays a crucial role in preventing further physical deterioration in these cases. Currently, PBD remains a debated practice in patients undergoing PD. Previous studies on PBD have shown conflicting results regarding the increased risk of infectious complications [14, 31, 40–42]. In our study, we did not find a significant increase in postoperative HAIs incidence associated with PBD. In a study of neoadjuvant chemotherapy (NAC) and postoperative complications of PD, the conclusion showed that biliary stent increased the occurrence of postoperative infectious complications and surgical site infections in neoadjuvant chemotherapy patients, which differed from the results of the present study [43]. Nadeem SO et al. found that patients who received NAC exhibited significantly increased growth of Gram-negative anaerobic bacteria [44]. However, the results of Gerke H et al’s study showed that there was no significant difference in the rate of wound infections, intra-abdominal abscesses and overall complications between the patients undergoing NAC with and without PBD [45]. In addition, Hamidi M et al. found that there is no difference in superficial surgical site infection and deep surgical site infection between preoperative biliary stenting (PBS) and no PBS in patient undergoing PD without NAC [46]. This suggests that the combined effect of PBD and NAC on postoperative infectious complications is still unclear and controversial. Further studies are needed in the future to explore this relationship. Our study did not take into account the effects of NAC due to the unavailability and absence of some data during data collection, suggesting that we need to further consider the role of NAC in the association between PBD and postoperative infection. However, subgroup analysis revealed that PBD in patients undergoing robotic-assisted PD was found to increase the risk of postoperative HAIs. Robotic-assisted PD provides advantages such as improved ergonomics, increased dexterity and stereotactic vision, enabling specialized surgeons in pancreatic surgery and minimally invasive techniques to benefit greatly [47]. While robotic-assisted PD greatly reduced postoperative HAIs in the non-PBD group due to technical advantages, it also magnified the impact of PBD on postoperative HAIs, resulting in a significant difference. Furthermore, the optimal duration for controlling the PBD-PD interval remains an unresolved issue [48]. Our present study demonstrated that an extended PBD-PD interval in patients with pancreatic cancer increased the risk of postoperative HAIs, which is consistent with findings from Sang et al. [49]. This can be attributed to the rapid progression of pancreatic cancer, where delaying surgery can compromise the efficiency of treatment [49, 50]. In our subgroup analysis of patients with bile duct cancer, BMI > 24 Kg/m^2^, and those undergoing robotic-assisted PD, we discovered that a short PBD-PD interval increased the risk of HAIs. A European multi-center study indicated that a PBD-PD interval exceeding 4 weeks resulted in fewer major complications, which aligns with the result of another retrospective study [48, 51]. Results from Sandini et al’s study showed that delaying surgery up to 1 month after biliary stenting may reduce major morbidity, procedure-related complications, and length of hospital stay, which differed from the results of the present study [48]. Their study had a higher median time and the operations in their study were performed for pancreatic cancer, whereas our study included not only patients with pancreatic cancer but also patients for other diseases and had a lower median time from stent placement to surgery. Additionally, Sandini et al’ s study took major complications as outcomes, and postoperative infections were not examined separately. However, our study found that delaying surgery after PBD may reduce the risk of postoperative HAIs in patients with cholangiocarcinoma. This suggests that we need to further explore the effects of drainage and surgical interval on postoperative infection in different diseases. Animal models have also demonstrated that it takes at least 4–6 weeks for significant liver functions to fully recover [52, 53]. Patients require sufficient recovery time PBD, and extent of liver function restoration depends on the timing of biliary decompression or the duration and severity of obstructive jaundice prior to decompression [54, 55]. As showed by previous study, POPF was the main complication after PD and an independent risk factor for infectious complications after PD [33]. In addition, Xiang C et al. found that the onset of grade B/C POPF may be triggered by infection [56]. This reflects the mutual influence of POPF and infection to a certain extent. Although there was a positive correlation between POPF and postoperative infection, it is difficult to distinguish the temporal sequence of POPF and postoperative infection because we were unable to confirm the occurrence time of pancreatic fistula in some of the patients during the process of data collection. Due to the inability to identify the temporal sequence of the occurrence for POPF and postoperative infection, we did not include POPF in the adjusted model. Therefore, we hope to overcome quality issues in data collection and investigate the effects of POPF and PBD on postoperative infection in future studies.

In real-world medical practice, the PBD-PD interval is influenced by various factors, including both patient’s physical recovery process and the availability of immediate surgical resources. These complexities surrounding PBD can be controversial, and therefore it is necessary to identify the suitable population for whom PBD is feasible. Based on the findings of our study, we recommend that the clinical PBD-PD interval should be extended, taking into consideration the patient’s physiological condition as well as healthcare resource availability. This approach facilitates patient recovery while reducing the risk of postoperative HAIs.

Our study also found that longer LOS, longer postoperative LOS, and more transfer of patients to ICU in the PBD group, which is consistent with previous studies [14, 22, 57]. Patients undergoing PBD usually need to wait until they have recovered before they can perform the surgery, thus extending the length of hospital stay. Our study did not reveal a significant association between PBD and the risk of postoperative complications and mortality. Nonetheless, our findings align with two reviews indicating that ENBD is significantly associated with increased overall postoperative complications and pancreatic fistula [58, 59]. This can be explained as that the inherent disruption of biliary tract sterility caused by endoscopic procedures, leading to an inflammatory response. The implement of PBD typically involves two main techniques: endoscopic biliary drainage (EBD) and percutaneous transhepatic biliary drainage (PTBD) [60]. With the advancement and refinement of endoscopic techniques, EBD has undergone further developments, manifesting as endoscopic biliary stenting (EBS) and endoscopic nasobiliary drainage (ENBD) [61]. The choice between these different types of biliary drainage depends on the specific clinical characteristics exhibited by the patient [25]. Additionally, the endoscope itself may inadvertently compromise the integrity of the tumor [14].

While previous studies have reported an increased incidence of postoperative HAIs and other complications following PBD [14, 31], we still recommend considering targeted PBD based on the patient’s individual circumstances and the cost-effectiveness of treatment. However, it is imperative to conduct further clinical trials to validate the correlation between PBD and the risk of postoperative HAIs and other complications in the future. By continuously refining our understanding of these factors, we can enhance patient outcomes and promote a more effective and targeted approach to infection prevention in the context of PD surgeries.

The study has several limitations. Firstly, this study retains a retrospective design despite its relatively large sample size, emphasizing the need for additional validation through prospective clinical trials. Secondly, certain patients were excluded from analysis due to multiple surgeries during their hospitalization, thereby impeding the evaluation of the impact of preoperative biliary drainage on this specific subgroup. Thirdly, further clinical assessment is warranted to assess the actual efficacy of the recommended interventions and antibiotic prophylaxis among patients undergoing PBD in reducing postoperative HAIs. Finally, we did not include the effect of NAC and POPF on postoperative HAIs in adjusted model due to the absence and unavailability of partial data during the collection of data. Anyway, our study offers valuable insights into the influence of PBD on HAIs and clinical outcomes in patients undergoing PD. The findings underscore the significance of PBD in the context of HAIs, but ongoing research is necessary to broaden our understanding of this phenomenon.

## Conclusion

Differing from results of other studies, there was no significant association between PBD and postoperative HAIs or other clinical outcomes. However, PBD was found to be significantly associated with an increased risk of extended LOS, postoperative LOS, and postoperative transfer of patient to ICU. Considering the patient’s economic and clinical factors, recommendations for implementing robotic-assisted PD and extending PBD-PD interval could potentially contribute to reducing the incidence of postoperative HAIs. Future research endeavors should focus on further exploring the optimal timing and techniques of PBD, as well as investigating other potential risk factors that might contribute to postoperative HAIs.

### Supplementary Information

**Supplementary Material 1.**

## Data Availability

The datasets used and analyzed during the current study are available from the corresponding authors on reasonable request.
