# Supplementary material for: The impact of preoperative biliary drainage on postoperative healthcare-associated infections and clinical outcomes following pancreaticoduodenectomy: a ten-year retrospective analysis
Source: BMC Infect Dis. 2024 Mar 28;24:361. doi: 10.1186/s12879-024-09246-8 (PMC10979617; doi:10.1186/s12879-024-09246-8)
Supplement: Supplementary file 1 — Supplementary Material 1. [file 12879_2024_9246_MOESM1_ESM.docx]

**Table S1.** HAIs rate and adjusted analyses assessing PBD for postoperative HAIs in subgroups.

| **Characteristics** |  | **infection** | **non-infection** | **adjusted analysis** | |
| --- | --- | --- | --- | --- | --- |
|  |  |  |  | **RR (95% CI)** | **P value** |
| **Primary disease** | |  |  |  |  |
| pancreatic cancer | |  |  |  |  |
|  | non-PBD | 33(5.7) | 545(94.3) | ref. |  |
|  | PBD | 22(8.7) | 231(91.3) | 1.683(0.949,2.984) | 0.075 |
| cholangiocarcinoma | |  |  |  |  |
|  | non-PBD | 41(13.2) | 270(86.8) | ref. |  |
|  | PBD | 35(10.6) | 294(89.4) | 0.813(0.498,1.325) | 0.406 |
| duodenal carcinoma | |  |  |  |  |
|  | non-PBD | 31(9.5) | 295(90.5) | ref. |  |
|  | PBD | 13(11.6) | 99(88.4) | 1.482(0.727,3.020) | 0.279 |
| ampullary carcinoma | |  |  |  |  |
|  | non-PBD | 12(11.9) | 89(88.1) | ref. |  |
|  | PBD | 11(9.5) | 105(90.5) | 0.743(0.305,1.810) | 0.513 |
| IPMN | |  |  |  |  |
|  | non-PBD | 5(3.0) | 160(97.0) | ref. |  |
|  | PBD | 1(9.1) | 10(90.9) | 8.511(0.58,124.808) | 0.118 |
| **BMI** | |  |  |  |  |
| <18.5 |  |  |  |  |  |
|  | non-PBD | 4(4.0) | 97(96.0) | ref. |  |
|  | PBD | 3(4.5) | 63(95.5) | 1.623(0.236,11.162) | 0.623 |
| 18.5-24 |  |  |  |  |  |
|  | non-PBD | 68(6.7) | 950(93.3) | ref. |  |
|  | PBD | 49(9.9) | 446(90.1) | 1.167(0.777,1.754) | 0.456 |
| 24-27 |  |  |  |  |  |
|  | non-PBD | 52(9.4) | 502(90.6) | ref. |  |
|  | PBD | 23(10.6) | 195(89.4) | 1.022(0.584,1.789) | 0.939 |
| ≥27 |  |  |  |  |  |
|  | non-PBD | 35(12.0) | 257(88.0) | ref. |  |
|  | PBD | 13(13.3) | 85(86.7) | 0.960(0.444,2.076) | 0.917 |
| **Age** |  |  |  |  |  |
| <65 |  |  |  |  |  |
|  | non-PBD | 109(7.8) | 1287(92.2) | ref. |  |
|  | PBD | 59(10.4) | 508(89.6) | 1.170(0.814,1.681) | 0.397 |
| ≥65 |  |  |  |  |  |
|  | non-PBD | 50(8.8) | 519(91.2) | ref. |  |
|  | PBD | 29(9.4) | 281(90.6) | 0.976(0.584,1.628) | 0.925 |
| **Type of operation** | |  |  |  |  |
| open |  |  |  |  |  |
|  | non-PBD | 131(9.5) | 1244(90.5) | ref. |  |
|  | PBD | 63(9.9) | 575(90.1) | 0.928(0.662,1.301) | 0.664 |
| robot |  |  |  |  |  |
|  | non-PBD | 20(4.4) | 438(95.6) | ref. |  |
|  | PBD | 16(9.9) | 145(90.1) | 2.174(1.011,4.674) | 0.047 |
| laparoscope |  |  |  |  |  |
|  | non-PBD | 7(7.0) | 93(93.0) | ref. |  |
|  | PBD | 8(12.7) | 55(87.3) | 3.403(0.846,13.685) | 0.085 |

Data are number of patients (%). PBD, preoperative biliary drainage; HAIs, hospital-associated infections; BMI, Body Mass Index; RR, risk ratios; 95% CI, 95% confidence interval.

**Table S2.** HAIs rate and adjusted analyses assessing type of PBD for postoperative HAIs in subgroups.

| **Characteristics** |  | **infection** | **non-infection** | **adjusted analysis** | |
| --- | --- | --- | --- | --- | --- |
|  |  |  |  | **RR (95% CI)** | **P value** |
| **Primary disease** | |  |  |  |  |
| pancreatic cancer | |  |  |  |  |
|  | non-PBD | 33(5.7) | 545(94.3) | ref. |  |
|  | PTBD | 19(8.6) | 202(91.4) | 1.680(0.924,3.055) | 0.089 |
|  | Stent | 2(10.0) | 18(90.0) | 1.875(0.408,8.611) | 0.419 |
|  | ENBD | 1(8.3) | 11(91.7) | 1.426(0.174,11.67) | 0.741 |
| cholangiocarcinoma | |  |  |  |  |
|  | non-PBD | 41(13.2) | 270(86.8) | ref. |  |
|  | PTBD | 29(11.5) | 223(88.5) | 0.883(0.526,1.483) | 0.639 |
|  | Stent | 4(8.7) | 42(91.3) | 0.647(0.217,1.929) | 0.435 |
|  | ENBD | 2(6.5) | 29(93.5) | 0.499(0.113,2.200) | 0.358 |
| duodenal carcinoma | |  |  |  |  |
|  | non-PBD | 31(9.5) | 295(90.5) | ref. |  |
|  | PTBD | 7(11.1) | 56(88.9) | 1.382(0.568,3.362) | 0.475 |
|  | Stent | 2(5.7) | 33(94.3) | 0.733(0.164,3.278) | 0.684 |
|  | ENBD | 4(28.6) | 10(71.4) | 4.241(1.177,15.287) | 0.027 |
| **BMI** | |  |  |  |  |
| 18.5-24 | |  |  |  |  |
|  | non-PBD | 68(6.7) | 950(93.3) | ref. |  |
|  | PTBD | 38(10.4) | 328(89.6) | 1.246(0.801,1.939) | 0.329 |
|  | Stent | 8(8.9) | 82(91.1) | 1.023(0.466,2.242) | 0.955 |
|  | ENBD | 3(7.7) | 36(92.3) | 0.838(0.248,2.831) | 0.777 |
| ≥24 | |  |  |  |  |
|  | non-PBD | 87(10.3) | 759(89.7) | ref. |  |
|  | PTBD | 28(11.3) | 220(88.7) | 1.009(0.621,1.640) | 0.972 |
|  | Stent | 3(7.0) | 40(93.0) | 0.527(0.156,1.778) | 0.302 |
|  | ENBD | 5(20.0) | 20(80.0) | 1.770(0.634,4.943) | 0.276 |
| **Age** | |  |  |  |  |
| <65 | |  |  |  |  |
|  | non-PBD | 109(7.8) | 1287(92.2) | ref. |  |
|  | PTBD | 45(10.6) | 381(89.4) | 1.180(0.794,1.753) | 0.413 |
|  | Stent | 8(8.9) | 82(91.1) | 1.029(0.475,2.227) | 0.943 |
|  | ENBD | 6(11.8) | 45(88.2) | 1.336(0.547,3.265) | 0.525 |
| ≥65 | |  |  |  |  |
|  | non-PBD | 50(8.8) | 519(91.2) | ref. |  |
|  | PTBD | 23(9.5) | 218(90.5) | 1.037(0.595,1.808) | 0.897 |
|  | Stent | 3(6.3) | 45(93.8) | 0.546(0.160,1.860) | 0.333 |
|  | ENBD | 3(14.3) | 18(85.7) | 1.527(0.421,5.545) | 0.52 |
| **Type of operation** | |  |  |  |  |
| open | |  |  |  |  |
|  | non-PBD | 131(9.5) | 1244(90.5) | ref. |  |
|  | PTBD | 46(9.6) | 434(90.4) | 0.903(0.621,1.313) | 0.593 |
|  | Stent | 10(9.9) | 91(90.1) | 0.881(0.440,1.763) | 0.72 |
|  | ENBD | 7(12.3) | 50(87.7) | 1.243(0.543,2.845) | 0.608 |

Data are number of patients (%). PBD, preoperative biliary drainage; PTBD, percutaneous transhepatic biliary drainage; ENBD, endoscopic nasobiliary drainage; HAIs, hospital-associated infections; BMI, Body Mass Index; RR, risk ratios; 95% CI, 95% confidence interval.

**Table S3.** HAIs rate and adjusted analyses assessing type of PBD for postoperative HAIs in subgroups.

| **Characteristics** |  | **infection** | **non-infection** | **adjusted analysis** | |
| --- | --- | --- | --- | --- | --- |
|  |  |  |  | **RR (95% CI)** | **P value** |
| **primary disease** |  |  |  |  |  |
| pancreatic cancer |  |  |  |  |  |
|  | non-PBD | 33(5.7) | 545(94.3) | ref. |  |
|  | ≤1 week | 3(5.4) | 53(94.6) | 0.985(0.287,3.377) | 0.981 |
|  | 1-4 week | 9(7.3) | 114(92.7) | 1.372(0.631,2.982) | 0.424 |
|  | ≥4 week | 10(13.5) | 64(86.5) | 2.992(1.358,6.592) | 0.007 |
| cholangiocarcinoma |  |  |  |  |  |
|  | non-PBD | 41(13.2) | 270(86.8) | ref. |  |
|  | ≤1 week | 12(19.0) | 51(81.0) | 1.695(0.814,3.528) | 0.158 |
|  | 1-4 week | 19(11.0) | 153(89.0) | 0.844(0.467,1.524) | 0.573 |
|  | ≥4 week | 4(4.3) | 90(95.7) | 0.292(0.100,0.853) | 0.024 |
| duodenal carcinoma |  |  |  |  |  |
|  | non-PBD | 31(9.5) | 295(90.5) | ref. |  |
|  | ≤1 week | 3(17.6) | 14(82.4) | 2.398(0.631,9.113) | 0.199 |
|  | 1-4 week | 5(9.4) | 48(90.6) | 1.125(0.410,3.091) | 0.819 |
|  | ≥4 week | 5(11.9) | 37(88.1) | 1.635(0.576,4.641) | 0.355 |
| **BMI** |  |  |  |  |  |
| 18.5-24 |  |  |  |  |  |
|  | non-PBD | 68(6.7) | 950(93.3) | ref. |  |
|  | ≤1 week | 6(7.1) | 78(92.9) | 0.858(0.355,2.076) | 0.735 |
|  | 1-4 week | 26(10.4) | 223(89.6) | 1.193(0.724,1.967) | 0.488 |
|  | ≥4 week | 17(10.5) | 145(89.5) | 1.286(0.724,2.283) | 0.391 |
| >24 |  |  |  |  |  |
|  | non-PBD | 87(10.3) | 759(89.7) | ref. |  |
|  | ≤1 week | 14(18.4) | 62(81.6) | 1.981(1.032,3.802) | 0.04 |
|  | 1-4 week | 12(7.7) | 143(92.3) | 0.644(0.335,1.237) | 0.186 |
|  | ≥4 week | 10(11.8) | 75(88.2) | 0.926(0.446,1.925) | 0.838 |
| **Age** |  |  |  |  |  |
| <65 |  |  |  |  |  |
|  | non-PBD | 109(7.8) | 1287(92.2) | ref. |  |
|  | ≤1 week | 14(14.6) | 82(85.4) | 1.660(0.889,3.099) | 0.112 |
|  | 1-4 week | 30(10.3) | 261(89.7) | 1.138(0.724,1.790) | 0.574 |
|  | ≥4 week | 15(8.3) | 165(91.7) | 0.958(0.534,1.719) | 0.887 |
| ≥65 |  |  |  |  |  |
|  | non-PBD | 50(8.8) | 519(91.2) | ref. |  |
|  | ≤1 week | 6(8.6) | 64(91.4) | 0.886(0.357,2.200) | 0.794 |
|  | 1-4 week | 10(6.9) | 134(93.1) | 0.694(0.334,1.445) | 0.329 |
|  | ≥4 week | 13(13.5) | 83(86.5) | 1.532(0.764,3.071) | 0.229 |
| **Type of operation** |  |  |  |  |  |
| open |  |  |  |  |  |
|  | non-PBD | 131(9.5) | 1244(90.5) | ref. |  |
|  | ≤1 week | 12(10.7) | 100(89.3) | 1.018(0.538,1.927) | 0.956 |
|  | 1-4 week | 31(9.6) | 291(90.4) | 0.894(0.581,1.375) | 0.61 |
|  | ≥4 week | 20(9.8) | 184(90.2) | 0.931(0.556,1.557) | 0.784 |
| robot |  |  |  |  |  |
|  | non-PBD | 20(4.4) | 438(95.6) | ref. |  |
|  | ≤2week | 5(16.7) | 25(83.3) | 3.058(1.178,7.940) | 0.022 |
|  | 2-4 week | 6(8.0) | 69(92) | 1.445(0.385,5.414) | 0.585 |
|  | ≥4 week | 5(8.9) | 51(91.1) | 1.713(0.573,5.122) | 0.335 |

Data are number of patients (%). PBD, preoperative biliary drainage; HAIs, hospital-associated infections; BMI, Body Mass Index; RR, risk ratios; 95% CI, 95% confidence interval.

**Table S4.** Logistic regression model analysis for assessing the impact of type of PBD on postoperative outcomes in patients undergoing PD.

| **Characteristics** |  | **positive rate (%)** | **adjusted analysis** | |
| --- | --- | --- | --- | --- |
|  |  |  | **aRR (95% CI)** | **P value** |
| **ICU, n (%)** |  |  |  |  |
|  | non-PBD | 635(32.3) | ref. |  |
|  | PTBD | 280(42.0) | 1.332(1.083,1.637) | 0.007 |
|  | Stent | 58(42.0) | 1.302(0.881,1.924) | 0.186 |
|  | ENBD | 35(48.6) | 1.489(0.896,2.476) | 0.124 |
| **Postoperative complications, n (%)** |  |  |  |  |
|  | non-PBD | 489(24.9) | ref. |  |
|  | PTBD | 149(22.3) | 0.901(0.720,1.129) | 0.365 |
|  | Stent | 36(26.1) | 1.073(0.716,1.607) | 0.734 |
|  | ENBD | 25(34.7) | 1.691(1.018,2.809) | 0.043 |
| **Pancreatic fistula, n (%)** |  |  |  |  |
|  | non-PBD | 208(10.6) | ref. |  |
|  | PTBD | 64(9.6) | 0.932(0.677,1.282) | 0.665 |
|  | Stent | 21(15.2) | 1.383(0.837,2.285) | 0.205 |
|  | ENBD | 15(20.8) | 2.181(1.187,4.007) | 0.012 |

PD, pancreaticoduodenectomy; PBD, preoperative biliary drainage; PTBD, percutaneous transhepatic biliary drainage; ENBD, endoscopic nasobiliary drainage; ICU, Intensive Care Unit; aRR, adjusted risk ratios; 95% CI, 95% confidence interval.

**Table S5.** Logistic regression model analysis for assessing the impact of PBD-surgery interval on postoperative outcomes in patients undergoing PD.

| **Characteristics** |  | **positive rate (%)** | **adjusted analysis** | |
| --- | --- | --- | --- | --- |
|  |  |  | **aRR (95% CI)** | **P value** |
| **ICU, n (%)** |  |  |  |  |
|  | non-PBD | 635(32.3) | ref. |  |
|  | ≤1 week | 56(33.7) | 0.958(0.666,1.380) | 0.82 |
|  | 1-4 week | 205(47.1) | 1.616(1.273,2.052) | <0.001 |
|  | ≥4 week | 112(40.6) | 1.248(0.936,1.663) | 0.131 |
| **Postoperative complications, n (%)** |  |  |  |  |
|  | non-PBD | 489(24.9) | ref. |  |
|  | ≤1 week | 44(26.5) | 1.084(0.747,1.572) | 0.671 |
|  | 1-4 week | 99(22.8) | 0.899(0.692,1.168) | 0.425 |
|  | ≥4 week | 67(24.3) | 0.992(0.730,1.347) | 0.957 |
| **Pancreatic fistula, n (%)** |  |  |  |  |
|  | non-PBD | 208(10.6) | ref. |  |
|  | ≤1 week | 20(12.0) | 1.160(0.698,1.930) | 0.566 |
|  | 1-4 week | 45(10.3) | 0.996(0.693,1.431) | 0.984 |
|  | ≥4 week | 35(12.7) | 1.256(0.840,1.877) | 0.267 |

PD, pancreaticoduodenectomy; PBD, preoperative biliary drainage; ICU, Intensive Care Unit; aRR, adjusted risk ratios; 95% CI, 95% confidence interval.

**Table S6.** Generalized linear regression model analysis for assessing the impact of type of PBD on postoperative outcomes in patients undergoing PD.

| **Characteristics** |  | **mean±SD** | **adjusted analysis** | |
| --- | --- | --- | --- | --- |
|  |  |  | **β (95% CI)** | **P value** |
| Hospital stay（days） |  |  |  |  |
|  | non-PBD | 23.0±9.7 | ref. |  |
|  | PTBD | 26.6±11.3 | 3.078(2.153,4.003) | <0.001 |
|  | Stent | 25.4±10.4 | 1.936(0.208,3.664) | 0.028 |
|  | ENBD | 27.0±12.5 | 3.179(0.833,5.524) | 0.008 |
| Postoperative hospital stay（days） |  |  |  |  |
|  | non-PBD | 15.9±8.4 | ref. |  |
|  | PTBD | 16.9±8.8 | 0.479(-0.298,1.256) | 0.227 |
|  | Stent | 16.6±8.3 | 0.205(-1.246,1.656) | 0.782 |
|  | ENBD | 18.1±9.2 | 1.425(-0.544,3.394) | 0.156 |

PD, pancreaticoduodenectomy; PBD, preoperative biliary drainage; PTBD, percutaneous transhepatic biliary drainage; ENBD, endoscopic nasobiliary drainage; 95% CI, 95% confidence interval.

**Table S7.** Generalized linear regression model analysis for assessing the impact of type of PBD on postoperative outcomes in patients undergoing PD.

| **Characteristics** |  | **mean±SD** | **adjusted analysis** | |
| --- | --- | --- | --- | --- |
|  |  |  | **β (95% CI)** | **P value** |
| Hospital stay**（**days**）** |  |  |  |  |
|  | non-PBD | 23.0±9.7 | ref. |  |
|  | ≤1 week | 25.2±9.1 | 1.518(-0.113,3.149) | 0.068 |
|  | 1-4 week | 27.2±11.8 | 3.495(2.409,4.582) | <0.001 |
|  | ≥4 week | 26.1±11.4 | 2.649(1.353,3.945) | <0.001 |
| Postoperative hospital stay**（**days**）** |  |  |  |  |
|  | non-PBD | 15.9±8.4 | ref. |  |
|  | ≤1 week | 16.8±7.8 | 0.296(-1.073,1.665) | 0.672 |
|  | 1-4 week | 17.2±9.3 | 0.601(-0.311,1.513) | 0.197 |
|  | ≥4 week | 16.6±8.3 | 0.293(-0.795,1.382) | 0.597 |

PD, pancreaticoduodenectomy; PBD, preoperative biliary drainage; 95% CI, 95% confidence interval.
